# Extraction, Characterization and Applications of Biopolymers from Sustainable Sources

**DOI:** 10.3390/polym18050581

**Published:** 2026-02-27

**Authors:** Elena Hurtado-Fernández, Luis A. Trujillo-Cayado, Paloma Álvarez-Mateos, Jenifer Santos

**Affiliations:** 1 Facultad de Ciencias de la Salud, Universidad Loyola Andalucía, Avda. de las Universidades s/n, Dos Hermanas, 41704 Sevilla, Spain; emhurtado@uloyola.es; 2 Departamento de Ingeniería Química, Facultad de Química, Universidad de Sevilla, c/Profesor García González s/n, 41011 Sevilla, Spain; palvarez@us.es

**Keywords:** biopolymers, green extraction, natural deep eutectic solvents (NADESs), ionic liquids, characterization, rheology, active packaging, biomedical applications, edible films, life cycle assessment (LCA)

## Abstract

Biopolymers from renewable sources are increasingly explored to reduce the carbon footprint of materials and mitigate plastic pollution. This review synthesizes the last five years of progress across the biopolymer value chain, comparing plant, microbial/fermentation, fungal, and marine/algal resources and critically assessing greener extraction and fractionation routes (ultrasound and microwave intensification, subcritical water, supercritical CO_2_ with co-solvents, ionic liquids, deep eutectic solvents including natural deep eutectic solvents, and enzymatic or bio-mediated processes). We emphasize yield-selectivity trade-offs, scalability, energy demand, and solvent recovery. Downstream, we summarize purification and performance tuning via crosslinking, derivatization, blending/plasticization, and nanocomposites, and we map advanced characterization to targeted functional properties to bridge processing choices with end-use performance. Applications are organized across food and agriculture, biomedical and pharmaceutical technologies, packaging, and cosmetics, with cross-cutting attention to safety and regulatory compliance, quality-by-design, techno-economics, and life-cycle assessment. Key bottlenecks are feedstock variability, viscosity and recyclability limitations of designer solvents, and persistent gaps in barrier and thermal properties versus petrochemical benchmarks, compounded by uneven composting and recycling infrastructure. Promising directions include low-viscosity or switchable solvents, data- and artificial intelligence (AI)-guided process optimization, engineered biopolymers, and circular end-of-life strategies that align material design with realistic recovery routes.

## 1. Introduction

Conventional petrochemical polyolefins (e.g., polyethylene, polypropylene) have become ubiquitous yet present critical sustainability challenges due to their extreme environmental persistence and resistance to degradation. These resilient plastics have a tendency to amass in landfill sites and within the confines of the ocean, where they act as microplastic pollutants, with a negligible proportion ever being subjected to recycling processes [1]. The inherent end-of-life limitations of these materials have created an urgent impetus to replace fossil-based plastics with biodegradable biopolymers. In contrast to inert polyolefins, a significant number of biopolymers are engineered to undergo safe degradation following utilization. Furthermore, these polymers can be designed with bioactive functionalities, thereby facilitating applications that exceed the scope of conventional plastics. For instance, biopolymers have been employed in biomedical tissue scaffolds and controlled drug delivery systems, which represent opportunities that are not available with conventional polyolefin materials. Furthermore, biopolymers have been used in soil-degradable agricultural mulch films and active food packaging that reduce persistent plastic waste [2,3].

The high carbon footprint of petrochemical polymers and persistent plastic pollution have driven a global shift towards bio-based, biodegradable polymers [4]. These biopolymers, which are large macromolecules produced by living organisms (such as polysaccharides, proteins and polyesters), offer the promise of reduced CO_2_ emissions and enhanced biodegradability at the end of their life. Unlike synthetic polymers, which have random structures, biopolymers often have well-defined architectures and low bond energies, making them more susceptible to environmental degradation [5]. These attributes make biopolymers an attractive alternative to non-renewable plastics in numerous applications. Indeed, global biopolymer production is rising steadily (projected to reach ~2.9 million tons by 2024) as industries seek sustainable, circular material solutions [3]. However, bringing biopolymers from the laboratory to the market requires overcoming significant challenges relating to feedstock supply, processing, performance and scalability.

In the extant literature, the terms biopolymer and bio-based polymer are sometimes used interchangeably, although they refer to different concepts. In accordance with prevailing terminology, biopolymers are defined as polymers that are biosynthesized by living organisms. From this standpoint, the term biopolymer encompasses several major families: (i) polysaccharides, such as cellulose, starch, chitin/chitosan, alginate, carrageenan, and pectin, which are abundant in terrestrial biomass, marine resources, and industrial by-products; (ii) proteins, including collagen/gelatin, whey and other dairy proteins, plant proteins (e.g., soy, pea, gluten), and emerging proteins from microbial or insect sources, which offer rich functionality through their amphiphilic character and hierarchical structures; (iii) biopolyesters synthesized by microorganisms or plants, most notably polyhydroxyalkanoates (PHAs), which can be biosynthesized and tailored through fermentation strategies; and (iv) other naturally occurring macromolecules such as lignin (aromatic biopolymer) and natural rubber, often exploited as functional components in blends and composites. Despite the marked differences in chemistry, supramolecular organization, and processing windows observed among these families, they share the common advantage of being derived from renewable biological resources. Furthermore, they can be engineered via a range of methods including extraction/fractionation, blending, plasticization, crosslinking, and composite design, thus allowing them to meet specific requirements for application.

Biopolymers can be produced from a wide range of natural feedstocks, including plant biomass, marine algae, and microbial and fungal cultures. The choice of feedstock has a significant impact on the properties of the polymer, its environmental impact and its economic viability. For example, carbohydrate-rich crops such as corn and sugarcane are well-established sources of bioplastics (polylactic acid, etc.) [6], whereas agricultural residues and organic waste are becoming recognized as low-cost, waste-derived materials [7]. Equally important is the extraction and processing methodology. Conventional polymer extraction often relies on harsh chemicals and energy-intensive processes, whereas green extraction technologies using benign solvents, enzymes or novel physicochemical methods are being developed to improve sustainability. Biopolymers often require downstream processing, such as purification and functionalization (e.g., chemical modification, blending, or plasticization), to give them the properties needed for their intended use. Advanced characterization techniques, ranging from spectroscopy and chromatography to microscopy and mechanical testing, provide a comprehensive understanding of structure–property relationships, thereby guiding quality control and material design.

This review provides a comprehensive examination of biopolymers from sustainable sources. Although this review primarily focuses on biopolymers biosynthesized by living organisms, selected bio-based polymers produced from bio-derived monomers (e.g., PLA and related systems) are also discussed when they are widely implemented in key application sectors, to provide a complete and practice-oriented perspective.

## 2. Sustainable Feedstocks and Supply

**Figure 1:**
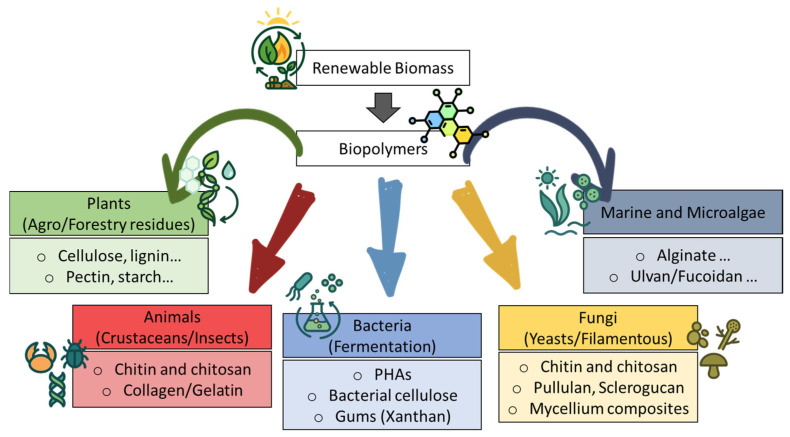
Representative feedstock sources of sustainable biopolymers, categorized by origin (plants, marine algae, animals, fungi, bacteria).

Securing adequate renewable feedstocks is a foundational step for sustainable biopolymer production. These feedstocks must be available in sufficient quantities and of an acceptable quality at a reasonable cost, ideally with minimal competition for food supplies [8]. Feedstocks can be derived from agricultural resources, such as dedicated crops or residues, or from waste materials, such as industrial by-products or municipal organic waste. Figure 1 illustrates the diverse natural origins of major biopolymers, ranging from plant and algal biomass to animal and microbial sources, reflecting the broad range of materials that can be used to produce bio-based polymers.

### 2.1. Plant-Based Feedstocks

Terrestrial plants are the most abundant source of biopolymers, primarily polysaccharides. Cellulose, the most abundant biopolymer on Earth, is a linear β-1,4-glucan that can be extracted from wood, grasses, crop residues (such as rice husks and corn stover), cotton linters, and even algae or bacterial cultures. Plant cellulose occurs as fibrous microfibrils with extensive hydrogen bonding, yielding a strong, insoluble framework that requires pre-processing for extraction [9]. Agricultural waste biomass (e.g., straw and husks) is an attractive source of cellulose, as it turns waste disposal problems into feedstock solutions. Hemicelluloses (e.g., xylans and glucomannans) and pectin are co-occurring plant polysaccharides [10]. Pectin can be recovered from fruit peels and apple pomace, and hemicelluloses can be obtained from wood or crop residues for use in biopolymer applications (e.g., pectin can be used as a gelling agent in foods and hemicellulose can be used to make films) [11,12]. Starch, a storage polysaccharide composed of amylose and amylopectin, can be obtained in large quantities from corn, potatoes, cassava and other crops. Native starch granules are semicrystalline and often require modification (e.g., acid thinning or dextrinization) to tailor their properties. Other plant-derived polymers include lignin (an aromatic polymer found in lignocellulosic biomass, which is usually produced as a by-product of pulping or cellulosic ethanol processes) and natural rubber (polyisoprene found in the latex of the Hevea tree), which are used in specialized applications in bio-based materials and elastomers [13,14]. Mucilages derived from aloe vera (*Aloe barbadensis*) leaves, chia (*Salvia hispanica*) seeds, and flaxseed (*Linum usitatissimum*) are notable plant-derived polysaccharide-rich materials that have garnered interest as sustainable biopolymer feedstocks. Chemically, these mucilages are heteropolysaccharides composed largely of neutral sugars (e.g., L-arabinose, D-xylose, D-galactose, D-mannose) and uronic acids (e.g., galacturonic and glucuronic acids) [15]. Aloe vera gel mucilage has been found to be particularly rich in acemannan, an acetylated mannose-containing glucomannan, along with pectic polysaccharides (rhamnogalacturonans and arabinogalactans) [16]. Chia and flaxseed mucilages have been found to contain significant arabinoxylan and pectic fractions. For instance, arabinose and xylose have been found to make up approximately 85% of chia seed mucilage monosaccharides, while flaxseed mucilage has been found to contain substantial proportions of xylose, galactose, rhamnose and galacturonic acid [17,18]. These water-soluble polymers have been shown to exhibit pronounced water-binding capacity and gel-forming ability, readily forming viscous gels or films [19]. The use of hydrocolloid properties in the production of edible films and encapsulation systems is facilitated by these substances, which function as natural thickeners, gelling agents and film-formers. Furthermore, their capacity for moisture retention and biocompatibility has led to their utilization in cosmetic applications, such as aloe vera-based hydrogels for skin moisturization [20]. Notably, integrating biopolymer production with existing agricultural and forestry operations (a biorefinery approach) can improve the sustainability of feedstocks by using residues and side streams rather than prime agricultural outputs [21].

### 2.2. Microbial and Fermentation-Derived Feedstocks

Microorganisms can synthesize a variety of polymers intracellularly or secrete them into the medium, making microbial fermentation a powerful method of producing biopolymers. Polyhydroxyalkanoates (PHAs), for example polyhydroxybutyrate (PHB), are a family of microbial polyesters produced by numerous bacteria as carbon storage granules. More than 30% of soil bacteria are capable of synthesizing PHAs under nutrient-limited conditions [22]. Industrial PHA production typically uses sugars or oils (e.g., corn glucose or waste oils) as feedstocks and can yield biodegradable thermoplastics with properties comparable to polypropylene [23]. Similarly, polylactic acid (PLA), while ultimately a synthetic polymer, is produced via the fermentation of sugars into lactic acid, followed by polymerization. PLA uses carbohydrate feedstocks such as corn or sugarcane, and large-scale production (hundreds of kilotons per year) has been achieved due to well-established agricultural supply chains. Bacterial fermentation also yields polysaccharides; for example, bacterial cellulose is secreted as a nanofibrillar network by *Komagataeibacter* species, producing a high-purity cellulose that is useful for medical and food applications [24]. Another example is xanthan, an exopolysaccharide secreted by *Xanthomonas campestris* during fermentation, which exhibits high viscosity at low concentrations and is widely used as a thickener and stabilizer in the food, pharmaceutical and cosmetic industries [25,26]. Beyond xanthan, fermentation can also yield other high-performance microbial exopolysaccharides from *Sphingomonas* spp., notably welan and diutan gums (sphingans). These polysaccharides show pronounced shear-thinning and strong viscosity retention under harsh conditions (e.g., elevated temperature and salinity), which has driven their industrial use as robust rheology modifiers in cement/concrete formulations and in enhanced oil recovery [27,28]. In addition, their tunable functionality through fermentation control and strain/process optimization makes them attractive candidates as thickeners/stabilizers in formulated products where process tolerance and long-term stability are required [29,30]. Finally, pullulan, a fungal biopolymer produced by *Aureobasidium pullulans*, forms edible films with high resistance to oxygen and heat. It is mainly used in antimicrobial coatings and films for food preservation [31,32].

It is important to note that fungi, in addition to bacteria, play a crucial role in the biosynthesis of degradable biopolymers, an aspect that will be detailed in the following section. Fungi (including yeasts and filamentous fungi) contribute to the supply of biopolymers in multiple ways. Similarly to crustacean shells, the cell walls of fungi contain chitin and β-glucans, which can be extracted as an alternative source of chitin that is free from marine allergens and available year-round from fungal fermentation [33]. Certain filamentous fungi secrete extracellular polysaccharides such as the aforementioned pullulan, scleroglucan and curdlan, all of which have niche uses in the food and industrial sectors. The mycelium of fungi can itself be processed into novel biopolymer-based materials [34]. For example, mats of mycelium (grown on lignocellulosic waste) can be dried and pressed to form mycelium composites, which are lightweight, biodegradable foams that are being explored for use in packaging and building materials [35]. Fungal biomass from fermentation (e.g., *Rhizopus* or mushroom cultivation waste) can therefore be used as a source of polymers and as a structural biomaterial [36]. Additionally, when genetically engineered, yeasts and filamentous fungi can produce polyesters such as PHA, or monomers such as lactic acid for PLA, thereby expanding the scope of microbial fermentation strategies. Proteins from fungal biomass (e.g., collagen-like proteins derived from fungi, or soy protein derived from plants as an analog) can also be formulated into bioplastics, although plasticizers and crosslinking are typically required due to brittleness.

### 2.3. Marine and Algal Feedstocks

The oceans provide a rich source of biomass for biopolymers, particularly seaweed-derived polysaccharides and chitin obtained from the waste of crustaceans. Brown seaweed (e.g., *Laminaria* and *Ascophyllum*) is harvested or cultivated as a source of alginate, which is an anionic polysaccharide composed of mannuronic and guluronic acid units. Alginate extracted from kelp or rockweed is widely used in food, pharmaceutical and tissue engineering products due to its gel-forming ability [37]. Red algae yield carrageenan and agar, which are sulfated galactans with gelling properties that are used extensively in food and microbiological media [38,39]. Chitin, the second most abundant polysaccharide in nature, is a structural polymer found in the exoskeletons of marine animals (such as crabs and shrimp) as well as in the cuticles of insects and the cell walls of fungi [40]. Industrially, chitin is obtained on a large scale from crustacean shell waste, a by-product of seafood processing. Following demineralization and deproteinization, chitin can be deacetylated further to produce chitosan, a valuable cationic biopolymer that is soluble in mild acid. Both chitin and chitosan are renewable marine biopolymers with applications in water treatment (binding pollutants) [41], agriculture (seed coatings and biopesticides) [42], biomedicine (wound dressings and drug delivery) [43], and cosmetics [44]. Seaweeds and microalgae also produce ulvan (from green algae) and fucoidan (from brown algae), which are sulphated polysaccharides being investigated for their bioactive properties [45,46]. An attractive feature of marine biomass is that it does not compete with terrestrial agriculture and can often grow rapidly (e.g., kelp farms) [47]. Certain strains of microalgae can be cultivated for polymer production [48]. For example, they can accumulate PHAs or produce bioplastic precursors under controlled growth conditions [49,50]. Overall, marine feedstocks expand the range of biopolymers while enabling the utilization of aquacultural or fishery waste, thus enhancing sustainability.

Ensuring a sustainable supply of all these feedstocks requires consideration of land/sea use, seasonality and logistics. Agro-residues and waste are particularly promising feedstocks as they do not require additional land cultivation and are often a negative cost due to the need for disposal. For instance, cheese whey and other food processing by-products can be used to feed microbial PHA production, creating value from waste. Policies such as subsidies for bioplastic feedstocks (e.g., U.S. Farm Bill initiatives) have begun to encourage farmers to supply materials such as corn starch for PLA. At the same time, competition with food use must be managed (the food vs. fuel debate extends to bioplastics), which has led to a shift towards using non-food crops (e.g., switchgrass for cellulose) or cultivating algae. Diverse sourcing also cushions against supply volatility. Ultimately, a combination of dedicated crops, industrial by-products and novel sources (e.g., algae and insects) is likely to be needed in order to robustly support a growing biopolymer industry [51]. By selecting and engineering feedstocks that are high-yielding and have a low environmental impact, researchers and industry aim to ensure that biopolymers deliver on their sustainability promises from the outset.

## 3. Green Extraction and Fractionation

**Figure 2:**
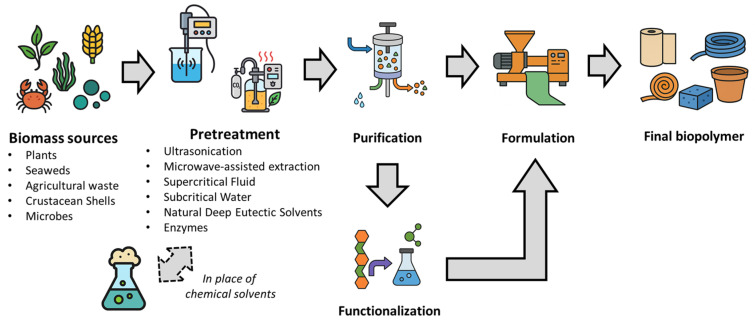
Integrated route for the production of sustainable biopolymers from biomass.

Once a suitable biomass feedstock has been obtained, the next challenge is to extract the target biopolymer efficiently and sustainably. While traditional extraction and fractionation methods can be effective, they often come with environmental and safety drawbacks, including the use of hazardous solvents, high energy demand and the generation of chemical waste. Green extraction approaches aim to minimize these issues by using environmentally friendly solvents and energy-efficient techniques, as well as recovering co-products [52]. Additionally, ‘fractionation’ refers to the integrated separation of the different polymeric components of biomass (for example, cellulose, hemicellulose and lignin from plant material), so that each fraction can be valorized [53]. There have been rapid advances in novel extraction technologies aligned with green chemistry principles in recent years [54,55]. Many biopolymers have traditionally been extracted using organic solvents or harsh chemicals [56]. For instance, PHAs are frequently recovered from bacteria by dissolving them in chlorinated solvents such as chloroform or dichloromethane, and then precipitating them with alcohol [57]. Chitin from shells is typically extracted using concentrated acids (to remove minerals) and bases (to remove proteins) at elevated temperatures [58]. While effective, these methods pose safety and disposal issues. Green solvent extraction aims to replace toxic reagents with safer alternatives or eliminate them entirely [58]. In the case of PHAs, bio-based solvents such as ethylic esters, dimethyl carbonate and 1,3-dioxolane have been investigated as less toxic alternatives to chloroform [59,60,61]. Using dilute sodium hypochlorite with surfactants can lyse cells and release PHAs without the need for halogenated solvents, although care must be taken to prevent polymer degradation [62]. For algal or plant polysaccharides, water-based extraction under controlled pH can often replace the use of organic solvents. For example, an extraction of brown algae Padina using a mineral acid (pH ~1.5), combined with centrifugation and filtration, was used to extract alginate, yielding significant polymer recovery [63]. The main disadvantage of solvent extraction (even with greener solvents) is the large volumes of solvent required, and the subsequent need to remove and recycle them. Thus, although solvent extraction is simple and widely used, it is increasingly combined with other techniques to improve efficiency. To provide an integrated overview of the pathway from raw biomass to functional biopolymers, Figure 2 illustrates the key stages of extraction, purification, functionalization, and formulation using green technologies.

### 3.1. Microwave- and Ultrasound-Assisted Extraction

These intensification techniques use physical energy to enhance the release of polymers from biomass. Microwave-assisted extraction (MAE) uses electromagnetic radiation (usually 100–1000 W at 915 MHz or 2.45 GHz) to quickly heat the moisture in the biomass [64]. This causes the cells to rupture and improves the penetration of the solvent. Microwaves can dramatically reduce extraction times from hours to minutes. For example, the optimal yield of carrageenan from the red alga *Mastocarpus* was achieved in just 6 min at 150 °C using microwave heating [65]. Microwaves provide fast volumetric heating, eliminating the thermal gradients associated with conventional heating methods. This results in uniform processing and often requires less solvent. This technique has been shown not only to accelerate extraction, but also to produce biopolymers with modified or improved properties (e.g., a lower molecular weight, which can be beneficial for certain applications) [66,67,68]. Ultrasound-assisted extraction (UAE) uses high-frequency sound waves (typically ~20–40 kHz) to generate cavitation in the extraction medium. The implosion of microbubbles near plant or algal tissue creates local shear forces that break cell walls and enhance mass transfer. UAE significantly reduces extraction time and can be performed at near-ambient temperatures, thus preserving heat-sensitive biopolymers [69]. Notably, the extraction of alginate from *Sargassum* seaweed using ultrasound was four times faster than the conventional method, with yields ~33% higher [70]. However, key parameters such as sonication time, intensity and temperature must be optimized to maximize yield without degrading polymer chains. Both MAE and UAE are scalable processes that have been implemented in pilot-scale systems for the extraction of pectin, alginate and starch, offering an energy-efficient alternative to lengthy reflux or maceration processes [71]. An additional environmentally friendly step is to couple these methods with membrane filtration or other non-solvent separation steps to further purify the extracts [72,73].

### 3.2. Subcritical and Supercritical Fluid Extraction

Subcritical water extraction (SWE) (also known as pressurized hot water extraction) involves the utilization of water at elevated temperatures (100–374 °C) and adequate pressure to ensure its liquid state is maintained [74]. In such conditions, the dielectric constant of water decreases, rendering it more non-polar and capable of solubilizing moderately hydrophobic biomolecules. This effect is analogous to the use of organic solvents, albeit with water as the medium. SWE has been successfully applied to the recovery of algal biopolymers such as fucoidan from brown seaweed, achieving higher yields (e.g., ~4.85% vs. 2.47%) than conventional methods [75]. The utilization of non-toxic solvents, expeditious kinetics, and frequently enhanced extract purity are among the salient advantages. In a similar manner, supercritical CO_2_ extraction (SC-CO_2_) is a widely utilized method for the extraction of non-polar compounds, such as lipids [74]. In addition, the application of SC-CO_2_ in combination with co-solvents for the extraction of polymers has emerged as a subject of interest, with examples including the extraction of PHA (polyhydroxyalcanoates) or polyhydroxybutyrate using supercritical CO_2_ with a co-solvent to enhance solubility [76]. CO_2_ above its critical point (approximately 31 °C, 74 bar) has been shown to act as a tunable solvent, exhibiting gas-like viscosity and liquid-like density [77]. This property affords it excellent penetration into biomass, facilitating straightforward separation through the simple process of depressurization to recover the solute. While SC-CO_2_ is considered ideal for the extraction of small molecules (e.g., oils and aromas), its utility for the extraction of high molecular weight polymers is limited unless a co-solvent (e.g., ethanol) is added, because pure CO_2_ is non-polar. It has been demonstrated that SC-CO_2_ has been successfully applied to the extraction of specialty biopolymers and the fractionation of lignocellulosic components (e.g., selectively removing lignin). A notable instance of the integration of methodologies is the utilization of supercritical water in conjunction with deep eutectic solvent (DES) additives, with the objective of enhancing the yield. A particular study involved the extraction of alginate and fucoidan from *Saccharina* algae, yielding alginate at a purity of approximately 28.1% and fucoidan at approximately 15%, through the incorporation of a DES within the hot water extraction process [78]. These approaches underscore the emergence of hybrid extraction techniques that employ a combination of thermal, chemical and physical mechanisms to enhance the recovery of biopolymers in a more environmentally friendly manner.

### 3.3. Deep Eutectic Solvents (DES) and Ionic Liquids (ILs)

A significant development in the field of green extraction is the utilization of designer solvents, such as ILs and DES, which possess the capability to selectively dissolve or separate biopolymers [79,80,81]. ILs are defined as thermally stable organic salts, frequently imidazolium-based cations with various anions. They have the capacity to dissolve otherwise recalcitrant polymers, such as cellulose and chitin, by disrupting hydrogen bonds. These enzymes have been employed in the fractionation of wood biomass, for instance, by facilitating the dissolution of cellulose and hemicellulose while preserving lignin, or vice versa [82]. However, it should be noted that many ILs can be costly, and their toxicity or difficulty in the complete removal from products should be considered. DES represent a novel class of environmentally friendly solvents, which are formed by combining two or more safe components (e.g., choline chloride and a natural acid or polyol) to create a eutectic mixture with a melting point that is substantially lower than that of the individual constituents. The use of inexpensive, biodegradable substances (e.g., choline chloride, urea, organic acids) by DES is a notable feature, and their adaptability to target specific interactions is a significant advantage [83]. In the context of chitin extraction, DES have demonstrated noteworthy efficiency. For instance, a choline chloride–lactic acid DES has been shown to yield chitin with a purity level comparable to that achieved by conventional acid/base methodologies when applied to shrimp shells [84]. Acidic DES effectively demineralizes shells by releasing H+ ions to dissolve calcium carbonate, while also solvating proteins via hydrogen-bond interactions, thus performing simultaneous demineralization and deproteinization. For protein-rich sources such as squid pens, alkaline DES (e.g., potassium carbonate with glycerol) has been shown to be more effective in the breakdown of proteins and the release of chitin [85]. The appeal of DES lies in their low volatility (no fumes), reusability, and often low toxicity (especially natural DES made from bio-based compounds). Furthermore, they facilitate one-pot processing, thereby obviating the necessity for separate acid and base steps, and instead permitting the attainment of chitin with a minimal requirement for further purification. Despite ongoing research, challenges remain in terms of both the scaling and complete removal of DES residues. Nevertheless, research is ongoing in order to optimize DES recycling and to assess their full life-cycle impacts. Beyond chitin, the effects of DES and ILs on lignocellulosic fractionation have been the subject of study, with the result that the selective extraction of lignin is now possible (lignin being soluble in certain aromatic ILs or DES) while cellulose remains intact [86]. This process facilitates the production of purified cellulose fibers and the separation of lignin for valorization, both of which are achieved under milder conditions than those employed in traditional pulping methods.

### 3.4. Enzymatic and Biological Extractions

Nature’s own catalysts (enzymes) have the capacity to perform highly selective extraction or modification of biopolymers under mild conditions. Enzymatic methods have been employed for the extraction of pectin (by means of pectinase, which liberates pectins from plant tissues) [87] and starch (by means of amylases, which facilitate controlled hydrolysis) [88]. In the process of chitin extraction, enzymes derived from microbes, such as proteases and chitin deacetylases, have been employed to substitute for the aggressive NaOH deproteinization method [89,90]. Remarkably, these enzymes have even been utilized to achieve partial deacetylation of chitin, resulting in the formation of chitosan. One such approach involves the solid-state fermentation of crustacean shells with specialized microorganisms that secrete organic acids and proteases in situ, thereby effectively decalcifying and removing proteins in a single bio-mediated process [91]. Such biological fractionation has the potential to significantly reduce chemical usage; however, the process of controlling this and preventing contamination can be challenging. Enzymatic hydrolysis is pivotal in the conversion of biomass to fermentable sugars for the purpose of microbial polymer production. For instance, the enzymatic hydrolysis of lactose in cheese whey to glucose and galactose has been shown to enhance downstream PHA yields with a reduced energy requirement when compared with acid hydrolysis [92]. In summary, the process of enzymatic extraction involves a compromise between the pursuit of speed and the attainment of specificity and eco-friendliness. This approach is frequently employed in conjunction with other methodologies, such as a mild enzyme treatment following a physical pretreatment, with the objective of optimizing yield.

### 3.5. Fractionation in Biorefineries

In contrast to the extraction of a single polymer and the subsequent discarding of the rest, contemporary biorefinery concepts seek to fractionate biomass into multiple valuable outputs, thereby enhancing overall sustainability. For instance, a lignocellulosic biorefinery might sequentially extract hemicelluloses (via hot-water or steam pre-treatment), delignify with an organic solvent or IL to isolate lignin, and leave behind nearly pure cellulose pulp for further processing [93]. In a similar manner, seaweed can be fractionated, with alginate extracted first by means of an alkaline treatment, and then, perhaps, cellulose recovered from the seaweed tissue. Proteins or minerals can also be extracted for other uses. In their 2015 study, Yuan and Macquarrie illustrated an approach by means of a comparison between direct alginate extraction and a biorefinery process on kelp [94]. The integrated process resulted in the production of multiple products and was found to be economically advantageous. The trade-off is process complexity, which must be justified by the value of additional co-products. Notwithstanding, the process of fractionation is congruent with the objective of zero-waste, as it guarantees the comprehensive utilization of a single organism (whether plant or algal in nature).

In order to provide a more comprehensive overview, a number of selected studies have reported quantitative comparisons that highlight the efficiency and scalability of different extraction methods. The extraction efficiency metrics demonstrate significant variations between methodologies, highlighting trade-offs in terms of yield and scalability. For instance, the use of DES in the extraction of chitin has yielded a yield of approximately 12–26%, accompanied by a purity level of 74–91%, in contrast to the conventional acid/alkali process, which has yielded a mere 6.5% yield with 91% purity [95]. In a similar manner, in the context of microbial biopolymer recovery, the utilization of a biodegradable solvent such as dimethyl carbonate (in conjunction with a cell-lysis pretreatment) has been shown to enhance PHA extraction yields from approximately 50% to approximately 80%, thereby achieving up to 94% recovery for PHBV under optimized conditions [62]. This recovery rate is comparable to that achieved by chlorinated solvent extraction, yet it is associated with a significantly reduced level of toxicity. These quantitative comparisons demonstrate that contemporary techniques, such as green solvents or assisted extraction, have the capacity to enhance product yield and reduce energy or chemical consumption. Nevertheless, it is imperative to emphasize that maintaining high efficiency at scale remains paramount, as lab-scale yields frequently diminish by at least 50% when scaled up [96,97].

## 4. Purification, Functionalization and Formulation

Biopolymers obtained from biomass frequently contain impurities, such as residual proteins, pigments, salts, or cell debris. Consequently, they may not immediately possess the performance characteristics required for end-use. It is imperative to emphasize that purification is of the essence in the removal of non-polymeric components, ensuring the consistency of quality. This is of particular significance in applications within the domains of food and biomedical sciences, where purity and safety are of the utmost importance. In addition to purification, a significant number of biopolymers profit from functionalization, which can be defined as a chemical or physical modification that tailors their properties (solubility, reactivity, mechanical strength, etc.). Following this process, the biopolymers are then formulated into usable material forms (films, fibers, hydrogels, composites). These steps serve to bridge the gap between isolated raw polymer and the final product, guided by a QbD mindset to achieve target specifications.

### 4.1. Purification

The purification process is contingent upon the polymer in question and the extraction method employed. In certain instances, the extraction process itself yields a relatively pure polymer (for instance, alginate precipitated with calcium forms a gel that can be washed and acid-exchanged to yield pure alginic acid). In other cases, significant downstream remediation is required. Chitin/chitosan provides an illustrative example: following initial acid/alkali treatment of shells, chitin may be decolorized using oxidizing agents (KMnO_4_, H_2_O_2_) to remove residual pigments and obtain a white product [98]. In a similar manner, cellulose extracted from lignocellulose may contain residual lignin or hemicellulose. The purification of cellulose pulp can be achieved through the implementation of additional bleaching steps, utilizing hydrogen peroxide or ozone, in conjunction with alkaline extraction methods [99]. In the case of PHA biopolymers, if hypochlorite digestion has been employed, it is essential that the polymer undergoes thorough washing and solvent reprecipitation in order to eliminate cell fragments and any potential degradation byproducts [100]. Contemporary methodologies are focused on the integration of extraction and purification processes. These approaches employ the use of DES or ILs, which facilitate the extraction and purification of the target polymer in a single step by selectively dissolving the impurities, rather than the polymer itself [101]. Membrane filtration is also a gentle purification tool [102]; ultrafiltration can separate polysaccharides from smaller molecules (salts, monosaccharides, phenolics) after an aqueous extraction, concentrating the polymer [103]. A salient point is that achieving high purity must be balanced with yield and eco-friendliness. For instance, pharmaceutical-grade chitosan might require extensive purification (e.g., dialysis, activated carbon treatments to remove endotoxins), whereas technical-grade chitosan for water treatment can be less pure. Adopting a QbD approach entails the identification of critical quality attributes (e.g., ash content, protein residue, heavy metal levels) for the polymer, contingent on its intended use. This identification is followed by the optimization of purification steps to ensure the reliable fulfillment of the aforementioned specifications. Regulatory standards, such as those pertaining to the purity of food additives or the ISO biocompatibility criteria for medical polymers, frequently stipulate the requisite purity levels. In essence, the process of purification serves to ensure the quality of the biopolymer is firmly established. This is achieved by the removal of undesirable substances, thereby producing a non-toxic material that is consistent in its composition and meets the stringent regulatory requirements specific to its intended application.

### 4.2. Functionalization

A significant number of native biopolymers, whilst intriguing, necessitate modification in order to enhance or adjust their functionality. The process of chemical functionalization has the capacity to introduce novel functional groups or to modify the structure of polymers. For instance, chitosan (obtained by deacetylating chitin) already represents a functionalization—converting acetylated amine groups to free amines, which renders it water-soluble in acidic conditions and highly bioactive [4]. Furthermore, the amino groups of chitosan enable cross-linking reactions, either with multi-functional electrophiles (e.g., glutaraldehyde, genipin) to form networks, or with polyanions (e.g., alginate) to form ionic complexes [104]. These modifications have a significant impact on the material’s properties, resulting in the formation of hydrogels or films with controlled swelling and mechanical strength. Cellulose is another biopolymer that has undergone extensive functionalization [105]. It can be esterified (e.g., cellulose acetate, nitrate), etherified (e.g., CMC—carboxymethyl cellulose), or graft-copolymerized to make thermoplastics or water-soluble derivatives. Such cellulose derivatives are ubiquitous; for instance, cellulose acetate is used in fibers and plastics, and CMC as a thickener in foods [106]. Starch is often subjected to chemical or physical modification in order to enhance its resilience and reduce its sensitivity to moisture [107]. Significant modifications include acid-thinning, which reduces the molecular weight to facilitate more efficient film formation, hydroxypropylation, which improves clarity and stability, and acetylation, which decreases the tendency for retrogradation. These modified starches find application in packaging films and biodegradable plastics, with properties that are tailored by the degree of substitution [108]. Proteins (e.g., gelatin, soy protein) can be crosslinked enzymatically (e.g., using transglutaminase) or chemically (e.g., glutaraldehyde) to improve water resistance and mechanical integrity [5]. Graft copolymerization represents a further versatile methodology, whereby synthetic polymer chains are grafted onto a natural polymer backbone (or vice versa) to create hybrid materials [109]. It is important to note that functionalization is not limited to chemical means; physical treatments such as gamma irradiation [110] or ultrasonication [111] have been shown to induce chain scission or branching, thereby altering molecular weight and consequently rheology and film properties. A particularly flexible strategy is to utilize blending and plasticization (a form of physical modification): combining a biopolymer with other polymers or plasticizers to achieve the desired behavior [112]. A common example of this is the blending of PLA with starch or PHA cost and improve biodegradability, or the addition of polyols (e.g., glycerol, sorbitol) as plasticizers to polysaccharide films in order to increase their flexibility [113]. Indeed, glycerol is among the most prevalent plasticizers employed in the manufacture of hydrophilic biopolymer films, a consequence of its capacity to intercalate between polymer chains, thereby enhancing mobility [114]. As a further illustration, nanocomposites, incorporating nanoscale fillers such as cellulose nanocrystals, clay platelets, or graphene oxide, can significantly enhance the mechanical and barrier properties of biopolymers [115]. Research has demonstrated that the incorporation of cellulose nanocrystals, in quantities equivalent to a few weight-percent of the total composition, into PLA or starch, can enhance the tensile strength of the resultant matrix. This process also concomitantly reduces the oxygen permeability of the material, without compromising its biodegradability [116]. In essence, the objective of functionalization strategies is to address the disparity in performance between natural polymers and conventional plastics. This is achieved by introducing characteristics such as toughness, stability, conductivity, antimicrobial activity, and other attributes as required.

Polysaccharide biopolymers possess abundant polar functional groups (e.g., –OH, –NH_2_, –COOH) that render them intrinsically hydrophilic and biodegradable [117]. These characteristics are associated with high moisture uptake and limited native mechanical strength. The functionalization of these reactive groups has been shown to be a means of tuning performance. For example, the grafting of hydrophobic or aromatic moieties onto a polysaccharide backbone has been demonstrated to reduce water solubility and strengthen barrier and mechanical properties, while cross-linking has been shown to increase rigidity and thermal stability. Such modifications must be consistent with the requirements of the specific application (for example, hydrophilic surfaces for cell-contact applications versus hydrophobic coatings for moisture resistance). Conversely, synthetic polymers such as polyethylene possess nonpolar, low-energy surfaces that inherently repel water. These surfaces often necessitate rigorous surface activation processes, such as plasma oxidation, to introduce functional groups [118]. Consequently, biopolymer functionalization employs native chemistry to satisfy specific application requirements, in contrast to conventional polymers, which necessitate more intensive treatment to modify their inert surfaces.

### 4.3. Formulation and Processing

In order to be of practical use, biopolymers must be formulated into final product forms, such as films, fibers, foams, or molded objects. It is fortunate that a significant proportion of biopolymers have the capacity to undergo processing techniques analogous to those employed in the synthesis of synthetic polymers. For instance, solution casting is a widely employed technique for the fabrication of polysaccharide and protein films. In this method, the polymer (frequently containing a plasticizer) is dissolved or dispersed in a suitable solvent, then cast and dried into a thin film. This method is suitable for laboratory-scale experimentation and a number of industrial packaging films, including biodegradable bags produced from starch/Polyvinyl alcohol (PVA) blends. The processing of biopolymers through thermoplastic deformation is possible under two conditions: firstly, if the biopolymers undergo a phase transition upon heating, and secondly, if they have undergone a process of modification that renders them thermoplastic. Thermoplastic starch, for instance, is produced by subjecting starch granules to heat and glycerol in an extruder, thereby generating a melt that can be transformed into films. Extrusion and injection molding are applied to a range of bioplastics, including PLA, PHAs, and certain protein bioplastics, with the addition of suitable additives. This enables the large-scale production of items such as utensils, packaging foams, and components. In one report, high-pressure homogenization was used to disperse starch nanoparticles in a starch film matrix. This process led to improved barrier properties (lower water vapor transmission and increased hydrophobicity) in the resulting films [119]. Furthermore, advancements in fabrication techniques are poised to assume a pivotal role. The additive manufacturing process known as 3D printing has been employed in the field of biopolymers, utilizing materials such as PLA in filament form and gelatin/alginate hydrogels (in bio-printing) to engineer bespoke shapes for biomedical scaffolds and prototypes. Electrospinning is another technique in which a polymer solution (for example, a collagen/chitosan/PVA blend) is electrified to produce nanofibers, which are useful for tissue engineering scaffolds with a high surface area. Coating technologies (e.g., dip-coating, spray-coating) are utilized for the purpose of applying biopolymer layers to surfaces. Examples of such applications include the use of edible coatings on food products (e.g., using chitosan or whey protein to extend shelf life) or biodegradable coatings on paper packaging to enhance water resistance. In the course of these processes, formulation encompasses not only the polymer itself but also additives, including plasticizers (as previously referenced), stabilizers (to prevent polymer degradation during processing; for example, antioxidants in PLA to slow thermal oxidation), and, in certain instances, active agents (for active packaging or biomedical function; for example, antimicrobial essential oils in a film or a drug loaded in a scaffold). The QbD approach in formulation involves the identification of critical process parameters (e.g., extrusion temperature, solvent evaporation rate) and critical material attributes (e.g., polymer molecular weight, moisture content) that affect product quality. For instance, elevated extrusion temperatures may induce thermal degradation of the biopolymer, resulting in a reduction in molecular weight and film strength. In such cases, QbD principles would mandate meticulous temperature control or online monitoring to ensure that the temperature remains within the established, acceptable range. The incorporation of such principles by manufacturers is intended to ensure consistent and reproducible outcomes, even in the presence of natural variability in feedstock.

The processing of natural biopolymers must accommodate their distinct thermal and moisture-sensitive properties. It is important to note that polysaccharides and proteins frequently absorb water and degrade at relatively low temperatures. Consequently, solvent-casting, extrusion or molding typically require the use of plasticizers, strict drying or pretreatment [5]. For instance, high humidity affinity has been demonstrated to cause agglomeration during processing, while excess heat or shear has been shown to induce hydrolytic breakdown. The blending and compounding of polymers with other polymers, plasticizers or fillers is a process employed to adjust viscosity, glass transition and mechanical strength. Furthermore, the application of chemical treatments during processing has been demonstrated to result in alterations to crystallinity or network formation. For instance, the grafting of cellulose with hydrophobic monomers has been shown to markedly decrease moisture uptake and raise thermal stability [5]. In comparison, petroleum-based polymers such as polyethylene exhibit significantly higher melt stability and minimal moisture affinity, rendering them more amenable to melting processes but less capable of biodegradation. It is imperative that formulation and processing conditions are meticulously regulated to ensure the attainment of the requisite mechanical strength, flexibility, and barrier properties for applications such as packaging films or biomedical scaffolds.

## 5. Application Landscape

**Figure 3:**
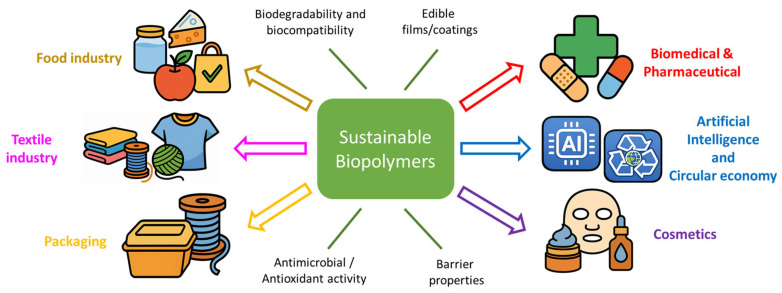
Functional properties of sustainable biopolymers and their main application areas.

The use of biopolymers from sustainable sources is driving innovation across a wide range of applications (see Figure 3). In this section, a survey of key application domains is presented, including food and agriculture, biomedical and pharmaceutical, packaging, and cosmetics. The representative uses, recent trends, and the advantages and challenges of biopolymers in each domain are highlighted. A unifying theme pervades this field, positing that biopolymers frequently confer unique functionalities (e.g., biodegradability, biocompatibility, renewable sourcing) that align with contemporary consumer and regulatory imperatives for sustainability and safety, even as performance optimization persists [120]. Recent advancements in the field have placed significant emphasis on multifunctional biopolymer systems that demonstrate potential for application across diverse sectors. A salient example of this is edible or biodegradable films that exhibit antimicrobial, antioxidant, or UV-shielding properties [121,122,123]. These films find dual use in both food and cosmetic packaging applications, underscoring the versatility of biopolymer systems in meeting diverse industrial and commercial needs. Furthermore, the integration of artificial intelligence (AI) with formulation design and high-throughput screening is progressively enhancing the simultaneous optimization of mechanical, barrier, and sensory properties, thereby expediting the translation of products from research and development to commercialization [124].

### 5.1. Food and Agriculture

Biopolymers are of significance in the field of edible films and coatings, thickeners, gelling agents, and delivery systems in foods and crops [125]. Polysaccharides (alginate, pectin, carrageenan, starch, cellulose derivatives) and proteins (gelatin, soy/whey) have been shown to stabilize emulsions, structure low-fat foods, and form active or intelligent packaging when combined with antimicrobials/antioxidants or pH-indicators [126,127]. The encapsulation of bioactive substances, including vitamins, polyphenols and probiotics, within alginate/pectin microgels or starch/chitosan particles has been shown to protect them and enable controlled release [128,129]. In the agricultural sector, biopolymer seed coatings and films have been shown to modulate moisture and fertilizer delivery, with the added advantage of biodegradation in soil [130]. The primary benefits include food-grade safety, compostability, and the valorization of by-products. The key challenges pertain to the development of moisture/oxygen barriers and water sensitivity, which are often addressed through the utilization of multilayers, hydrophobic coatings, or nanofillers (e.g., nanocellulose/clays). Integration with composting or anaerobic digestion is imperative to realize end-of-life benefits.

### 5.2. Biomedical and Pharmaceutical

The utilization of natural and bio-based polymers has emerged as a pivotal aspect in the field, facilitating the development of biocompatible, resorbable devices and drug carriers [131,132]. A plethora of materials are utilized in the fabrication of wound dressings, hemostats, tissue-engineering scaffolds, sutures, and long-acting depots. These materials include, but are not limited to, chitosan, alginate, hyaluronic acid, collagen/gelatin, bacterial cellulose, and bio-polyesters (PLA/PLGA, PHAs). It is evident that hydrogel and nanoparticle platforms (for example, chitosan or PLGA) provide tunable mechanics and controlled release [133]. Furthermore, bioinks based on alginate/gelatin-methacrylate support 3D bioprinting [134]. The process of translation is contingent upon adherence to ISO 10993 biocompatibility standards, the implementation of Good Manufacturing Practice/Quality by Design (GMP/QbD) control of molecular weight, the presence of endotoxins, residuals, and the use of predictable degradation kinetics (e.g., acid build-up, local pH) [135]. Open challenges include batch variability, sterilization without property loss, and robust scale-up; fast-moving fronts are stimuli-responsive/smart hydrogels, conductive biopolymer composites, and transient/fully resorbable electronics.

In the medical field, biopolymers are selected and modified to achieve a balance between biocompatibility, biodegradation rate, and mechanical support. Natural polymers (proteins such as collagen/gelatin and polysaccharides such as alginate/chitosan) inherently possess cell-friendly surfaces and yield non-toxic, resorbable byproducts [136]. These materials have the capacity to replicate the mechanical properties of tissue and can be used to facilitate the infiltration of pores or hydrogels by cells, as well as to enable the diffusion of drugs. As previously described, natural biopolymers offer intrinsic hydrophilicity and biodegradability, which are tuned to match target tissues. The process of functionalization serves to further regulate the degradation kinetics and biological interactions. In contrast, synthetic polymers such as polyethylene are inert and non-degrading. They provide long-term structural integrity but do not resorb and may elicit foreign-body reactions [137]. Consequently, biomedical applications frequently favor biopolymers, whose thermal and mechanical behavior can be engineered for temporary implants or drug carriers, while synthetic polymers remain for permanent, load-bearing devices

### 5.3. Packaging

Biopolymers can be categorized into two distinct groups, namely rigid and flexible formats [138]. The former encompasses materials such as PLA and PHAs, while the latter includes TPS-PBAT/PLA blends, cellulose-based films and coated papers [139,140]. In many short-lived applications, strength and clarity are competitive; however, shortcomings such as heat resistance, moisture/oxygen barrier, and brittleness can be mitigated through the use of crystallization control, compatibilized blends, multilayers, and nano-reinforcements (e.g., nanocellulose, clays). Active packaging (antimicrobial/antioxidant) and paper coatings (chitosan/PLA/PHA) have been shown to expand functionality. The final stage of the waste management process is pivotal in determining the sustainability of the material. Industrial composting favors the degradation of PLA and thermoplastic polyurethane (TPU) in the presence of certain polyhydroxy acids (PHA), which are capable of biodegradation in marine environments. The progression of methodologies pertaining to the sorting and labeling of materials, in conjunction with the chemical recycling process (for instance, the depolymerization of PLA) and the integration of LCA-guided design, is of paramount importance. Whilst financial cost and supply-chain scalability continue to represent significant constraints, these issues are being addressed through the development of larger manufacturing facilities, the use of waste-stream feedstocks, and the implementation of policy incentives.

The materials used in the production of packaging films must exhibit three key properties: good mechanical strength, gas-barrier performance, and controlled moisture permeability. Biopolymer films, for instance starch, cellulose and gelatin, characteristically manifest robust intermolecular bonding, thereby conferring remarkable oxygen and gas barrier properties, while concomitantly exhibiting elevated hydrophilicity. For instance, starch- or cellulose-based films have been observed to demonstrate inadequate water vapor resistance, while gelatin networks exhibit swelling and softening in humid environments [141]. In order to meet the requirements of the packaging, a range of strategies are applied during processing and functionalization. Plasticizers and blending are used to improve flexibility, while crosslinking or hydrophobic coatings are employed to reduce moisture uptake. For instance, the use of enzymatic or chemical crosslinking agents, such as genipin or polyphenols, has been demonstrated to enhance the hydrophobicity of protein films and reduce their water vapor permeability [142]. Furthermore, the incorporation of waxes, essential oils, or nanoclays into composite materials has been demonstrated to enhance their barrier properties. In contrast, synthetic polyolefins such as polyethylene inherently exhibit resistance to water vapor and provide robust mechanical properties. Therefore, in order to achieve performance comparable to that of polyethylene with biopolymers, targeted formulation and processing is required in order to compensate for their inherent moisture sensitivity.

### 5.4. Cosmetics and Personal Care

Bio-derived polymers have been demonstrated to deliver rheology, film-forming properties, moisture retention, and delivery in skin and hair products [143,144]. Hyaluronic acid (fermentation-grade) provides humectancy [145]; xanthan and cellulose derivatives regulate viscosity [128,146]; pullulan and chitosan form breathable films for tightening/conditioning; guar derivatives act as cationic conditioners in shampoos [44]. The utilization of cyclodextrins, lipid/biopolymer nanoparticles, and alginate/chitosan microcapsules facilitates the stabilization and controlled release of fragrances and actives [147]. The use of biopolymer beads and dissolvable microneedles facilitates microplastic-free exfoliation and transdermal delivery [44]. The acceptance of regulatory standards is facilitated by the existence of extensive safety records; however, it is imperative to consider the specifications for cosmetic grade, including metals, allergens, endotoxins and microbiology, in addition to ensuring consistent supply. Emerging research directions include the use of marine/fungal exopolysaccharides, biodegradable film-formers for long-wear makeup, and bio-manufactured polymers tailored for sensorial performance.

### 5.5. Textile Industry

The increasing demand for sustainable materials in the textile industry has prompted research into renewable biopolymers as an alternative to conventional synthetic polymers [148]. The inherent biodegradability, biocompatibility and low toxicity of these polymers renders them ideal for a variety of textile applications, ranging from fiber production to finishes and functional coatings. The most representative biopolymers in this sector include poly(lactic acid) (PLA), polyhydroxyalkanoates (PHA), cellulose (and its derivatives), and chitosan, amongst others [149,150]. Polymeric Lactic Acid (PLA) is typically is utilized in fibers and non-woven fabrics due to its compostability and adequate mechanical properties. Nevertheless, it has limited thermal resistance and stability, which frequently necessitates copolymerization or blending with other polymers to enhance the final performance [151]. PHA offer moderate mechanical strength, stability against UV radiation and hydrolytic degradation, and are compostable, allowing them to be processed into fibers (including electrospun nanofibers for medical and filtration applications) [152]. However, the most common counterpart, PHB, is fragile in nature, which limits its direct use in textiles without modifications or adequate plasticization [153]. Cellulose, a component of natural fibers (e.g., cotton) and regenerated fibers (e.g., viscose, lyocell), is both abundant and biodegradable. This property endows fabrics with enhanced breathability and moisture absorption capabilities. Finally, chitosan is employed as an antimicrobial and anti-odor finishing agent in textiles due to its biocompatibility and antimicrobial capacity. However, its low solubility at neutral pH and poor durability on the fiber restrict its application on an industrial scale. It is important to note that other biopolymers, such as marine alginates or natural proteins (e.g., silk, collagen), are also being explored in the form of specialized fibers or functional coatings [154]. This demonstrates the potential of biopolymers materials for developing advanced and sustainable textiles.

### 5.6. AI-Guided Optimization and Circular Economy Strategies in Biopolymers

Recent advances have demonstrated the potential of artificial intelligence (AI) and machine learning (ML) as effective tools for accelerating sustainable polymer development [155,156]. Machine-learning models that have been trained on polymer structure–property databases have been shown to be capable of predicting properties and identifying complex correlations at a faster rate than traditional methods [157]. For instance, AI-driven molecular modeling has the capacity to screen vast libraries of biopolymer monomers and propose new structures that are optimized for biodegradability and performance [124,158]. The utilization of generative models and predictive machine learning has already been demonstrated in the design of bio-based plastics with tailored mechanical and degradation profiles [159]. Within the domain of manufacturing, machine learning (ML) is enabling real-time process control. Specifically, models have the capacity to ingest sensor data during fermentation, extrusion or molding processes, thereby enabling the prediction of variability in feedstock or process drift [156]. Consequently, these models have the capacity to adjust parameters proactively. The implementation of such “smart” optimization strategies has been demonstrated to be effective in stabilizing extrusion lines operating at elevated levels of recycled content [160]. This approach has been demonstrated to contribute to the minimization of waste and energy consumption [156].

The concept of a circular economy is also being incorporated into the field of biopolymer innovation [161]. Researchers emphasize feedstock valorization and waste integration by using non-food biomass and agricultural residues as polymer precursors. For instance, cellulose and starch extracted from crop or food-processing waste are converted into compostable packaging films, thereby transforming waste streams into high-value materials [162]. Design for recyclability is also an active focus in this field. Novel bio-polyesters are engineered to be chemically recyclable back to monomers, thus closing the loop. Akinsemolu et al. observe that the effective implementation of biopolymer recycling technologies necessitates their integration with circular-economy models and the facilitation of collaboration among stakeholders, thereby ensuring the operationalization of closed-loop systems [163]. It is evident that artificial intelligence is beginning to support these circular strategies as well: The use of machine learning (ML)-augmented life-cycle assessment tools facilitate the prediction of the environmental impacts of polymer variants, thereby assisting in the design of materials that are characterized by reduced carbon emissions or enhanced recyclability [164]. The integration of AI and circularity enables biopolymer innovation to be “sustainability-aware,” whereby materials are designed with both performance and end-of-life considerations in mind. Furthermore, production processes are streamlined for resource efficiency and reuse.

## 6. Conclusions and Perspectives

This review has summarized the main advances in sustainable biopolymers, spanning renewable sources and recovery routes, structure–property relationships, and representative application domains. The field exhibits notable strengths, including access to renewable feedstocks, chemical and functional versatility, and the potential for performance enhancement through blending, crosslinking, and composite design. Concurrently, several limitations continue to constrain its extensive implementation, including feedstock variability, the scalability and cost of green processing, performance trade-offs, and the necessity for standardized testing and robust long-term validation under realistic use conditions. It is important to note that there are still key unanswered questions regarding harmonized definitions and metrics (e.g., bio-based content vs. biodegradability/compostability), reliable end-of-life behavior across environments, safe-by-design selection of additives and modifiers, and the integration of techno-economic and life-cycle perspectives to ensure that improved material performance translates into verifiable sustainability gains.

Biopolymers are evolving rapidly from niche materials into strategic platforms for more sustainable products in a variety of sectors. However, their wider adoption requires overcoming a range of interconnected challenges relating to feedstocks, processing, performance, safety and end-of-life issues. The first critical barrier is variability in feedstocks. As biopolymers are derived from living systems and biomass, their composition, molecular weight, impurities and functionality can differ depending on the species, season and processing history. This directly affects reproducibility, processability and final performance. Progress will depend on improved fractionation and purification strategies, tighter specification windows, and predictive quality control approaches that enable consistent ‘materials-by-design’ development.

A second major hurdle is implementing genuinely sustainable, scalable processing. While many extraction and modification routes are described as environmentally friendly, industrial implementation is often limited by solvent recovery, water usage, energy consumption, and the production of additional waste. Future research should therefore focus on process intensification, solvent and water recirculation, continuous manufacturing and robust scaling-up protocols that preserve functionality while achieving competitive yields and costs. In parallel, accelerating adoption requires a deeper mechanistic understanding of structure–property–function relationships across length scales. Many biopolymer-based materials exhibit a hierarchical organization which governs rheology, barrier performance, mechanical integrity and functional outcomes. Integrating advanced characterization with modeling and data-driven optimization can reduce the need for empirical trial and error and improve formulation efficiency.

Beyond performance in short-term laboratory tests, wider application depends on durability under realistic service conditions, including exposure to humidity and temperature cycling, UV radiation, mechanical fatigue, and repeated cleaning or sterilization. Standardized aging protocols and application-relevant metrics are needed to bridge the gap between bench-scale demonstrations and reliable long-term use. Material engineering strategies such as controlled crosslinking, compatibilization, blending and composite/coating design will be essential but must be aligned with safe-by-design and end-of-life considerations to avoid compromising recyclability or biodegradation pathways.

Finally, the field must converge on clearer definitions and harmonized evaluation frameworks, particularly for distinguishing bio-based content from biodegradability/compostability and for reporting end-of-life performance across relevant environments (industrial compost, home compost, soil and aquatic conditions). For applications involving direct or indirect human exposure, rigorous assessment of toxicology, migration, allergenicity and additive safety is indispensable, together with regulatory alignment. Crucially, sustainability claims must be backed up by an integrated techno-economic analysis and life-cycle assessment because the overall benefit of a given biopolymer system depends on the context and can be reduced by low yields, energy-intensive processing or inadequate end-of-life management. Addressing these priorities will require coordinated, interdisciplinary efforts connecting polymer science, bioprocess engineering, formulation and interface science, safety assessment and industrial ecology, ultimately enabling the development of biopolymer-based materials that deliver reliable performance at scale with verifiable environmental advantages.

## Data Availability

No new data were created or analyzed in this study. Data sharing is not applicable to this article.

## Abbreviations

The following abbreviations are used in this manuscript:

NADES	Natural deep eutectic solvents
LCA	Life cycle assessment
QbD	Quality by Design
PHAs	Polyhydroxyalkanoates
PHB	Polyhydroxybutyrate
PLA	Polylactic acid
MAE	Microwave-assisted extraction
UAE	Ultrasound-assisted extraction
SWE	Subcritical water extraction
SC-CO_2_	Supercritical CO_2_ extraction
DES	Deep eutectic solvent
ILs	Ionic liquids
CMC	Carboxymethyl cellulose
PVA	Polyvinyl alcohol
PLGA	Poly(lactic-co-glycolic) acid
GMP	Good manufacturing practice
TPS-PBAT	Thermoplastic starch blended with polybutylene adipate-co-terephthalate
TPU	Thermoplastic polyurethane
